# A Novel Approach to Surface Roughness Virtual Sample Generation to Address the Small Sample Size Problem in Ultra-Precision Machining

**DOI:** 10.3390/s24113621

**Published:** 2024-06-04

**Authors:** Ruilin Liu, Wenwen Tian

**Affiliations:** 1 School of Mathematics and Physics, Lanzhou Jiaotong University, Lanzhou 730070, China; liuruilin@lzjtu.edu.cn; 2 School of Automation and Electrical Engineering, Lanzhou Jiaotong University, Lanzhou 730070, China

**Keywords:** ultra-precision machining, surface roughness, small sample size, virtual sample generation, smart manufacturing

## Abstract

Surface roughness is one of the main bases for measuring the surface quality of machined parts. A large amount of training data can effectively improve model prediction accuracy. However, obtaining a large and complete surface roughness sample dataset during the ultra-precision machining process is a challenging task. In this article, a novel virtual sample generation scheme (PSOVSGBLS) for surface roughness is designed to address the small sample problem in ultra-precision machining, which utilizes a particle swarm optimization algorithm combined with a broad learning system to generate virtual samples, enriching the diversity of samples by filling the information gaps between the original small samples. Finally, a set of ultra-precision micro-groove cutting experiments was carried out to verify the feasibility of the proposed virtual sample generation scheme, and the results show that the prediction error of the surface roughness prediction model was significantly reduced after adding virtual samples.

## 1. Introduction

Smart machining has tremendous potential and is becoming a cornerstone of the new generation of high-value precision manufacturing technologies, in line with the advancements of Industry 4.0 concepts [1]. Ultra-precision machining (UPM) involves the manufacture of a high-quality surface at a nanometric surface roughness level. The effects of various factors on surface roughness formation have been widely studied. These factors generally include machine tools, cutting conditions, tool geometries, environmental conditions, material properties, chip formations, tool wear, vibrations, etc. [2]. In most cases, the surface roughness of mechanical products is one of the main technical requirements and is widely used as an indicator of product quality [3]. Generally, the surface roughness is controlled within the required threshold range by selecting a suitable combination of cutting parameters from time-consuming and laborious cutting tests based on manual experience. During the machining process, accurate prediction and control of surface roughness can improve machining efficiency and reduce machining costs [4].

In recent years, with the development of information and sensor technologies, as well as their widespread application in manufacturing, surface roughness prediction methods have been broadly divided into two categories: model-based and data-based. Theoretical modeling of surface roughness is realized through in-depth analysis after fully considering the kinematics, dynamics, and physical phenomena observed during the cutting process. For example, a model-based simulation system for the analysis of surface roughness generation in ultra-precision diamond turning was presented in [5]. After carefully analyzing the influencing factors of diamond-turned surface morphology, a comprehensive model for predicting the surface roughness achieved by single-point diamond turning (SPDT) was established in [6]. To predict surface roughness in the SPDT process, a prediction model incorporating both actual tool-work vibrations and material swellings was presented in [7]. An improved surface roughness prediction model was proposed to accurately estimate surface roughness in diamond turning of Al 6061. The model takes into account a number of factors, including the minimum undeformed chip thickness, as well as the material’s plastic side flow, elastic recovery, and precipitation effects [8]. Considering the complexity of the cutting process, despite a great deal of effort devoted to studying the factors influencing surface roughness generation in UPM, some of the physical mechanisms involved in material removal are still not fully understood, which hinders further improvements or assurance of surface quality [2]. In this regard, it is difficult to establish a theoretical model of surface roughness that is accurate enough to account for these factors.

Data-driven techniques have evolved rapidly over the past two decades, aiming to make full use of the large amount of available process data to obtain useful information, and have been widely applied to industrial process monitoring and control [9]. With the emergence of artificial intelligence technology in recent years, data-driven methods for predicting surface roughness have become increasingly popular among scholars and engineers. In the process of data-based surface roughness modeling, the main sources of input data mainly include process parameters, additional sensor signals, and built-in signals from CNC machines, with additional sensors effectively capturing the dynamic changes in surface roughness. Among the data-driven methods, a hybrid Bayesian inference-based hidden Markov model and a least squares support vector machine model for evaluating the surface roughness of hard turning have been proposed, which use the multi-directional fusion features extracted from monitoring signals by independent component analysis and singular spectrum analysis as model inputs [10]. To address the problem of small sample size, a grey online modeling surface roughness monitoring system based on force signals was proposed in [11]. In order to improve the prediction accuracy of surface roughness during milling, vibration signals of the workpiece, fixture, and spindle are used as monitoring information, and Bayesian linear regression is employed to construct a surface roughness prediction model [12]. Aiming at the problems of time-consuming training and low prediction accuracy in traditional surface roughness virtual metrology models, a novel fuzzy echo state broad learning system (FESBLS) was proposed in [13]. The results showed that the proposed FESBLS outperformed other models in improving prediction performance.

In the actual machining process, the small sample problem has been one of the key factors affecting the prediction accuracy of surface roughness. To improve accuracy, a prediction approach known as a predicted point-oriented local linear estimator has been proposed, which consists of two prediction stages: the first stage involves generating effective pseudo-data, and the second stage involves focusing on the final prediction based on a large number of pseudo-samples [14]. To address the problems of high collection costs, unbalanced categories, and complicated data distribution for part surface roughness samples, a novel data augmentation method based on CoralGAN for surface roughness prediction was presented in [15], where CoralGAN can learn the complicated sample data distribution and generate sample data. In order to achieve small sample enhancement of surface roughness, an interpolation-based virtual sample generation scheme using cutting parameters as input was designed in [16], which improves the model prediction accuracy to a certain extent. Moreover, a partial Bayesian co-training method for semisupervised virtual metrology scenarios was developed in [17], where labeled data are expensive to acquire and unlabeled data are abundant.

As mentioned above, it would be interesting to build an accurate surface roughness prediction model with low cost. However, data-based surface roughness prediction has rarely been investigated in UPM due to the small sample size problem and the requirement for prediction accuracy. A large number of studies have shown that the virtual sample generation (VSG) technique will be an effective solution to the small sample problem, and it is widely used in petroleum, chemical, and other process industries [18,19,20,21,22].

In the present work, we addressed the small sample problem of surface roughness in the UPM process by developing a novel surface roughness virtual sample generation scheme. First, feature extraction and selection were performed on the machining process monitoring signals. Then, particle swarm optimization (PSO) was used in conjunction with a broad learning system to generate surface roughness virtual samples. In addition, to validate the feasibility of the proposed scheme, a set of UPM groove-cutting experiments were conducted. The experimental results indicated that incorporating appropriate virtual samples significantly improved model prediction accuracy, proving to be more effective than solely enhancing small samples based on cutting parameters.

The main contributions of this work are summarized as follows:

- A novel scheme for generating virtual samples of surface roughness is proposed, which combines PSO and a broad learning system to increase the quality of virtual samples.
- Wavelet packet transformation is used to extract the features of the monitoring force signals recorded during the cutting process, and then the maximal information coefficient is used for feature selection.
- A set of ultra-precision groove-cutting experiments was conducted to verify the feasibility and superiority of the proposed small sample enhancement scheme. The results were compared with those obtained from generating virtual samples using cutting parameters.

The remainder of this article is structured as follows. Section 2 presents the proposed surface roughness virtual sample generation scheme. Section 3 describes the experimental design. The results and discussion are presented in Section 4. Finally, Section 5 provides the conclusions and insights into future trends.

## 2. Surface Roughness Virtual Sample Generation

### 2.1. Broad Learning System

**Figure 1:**
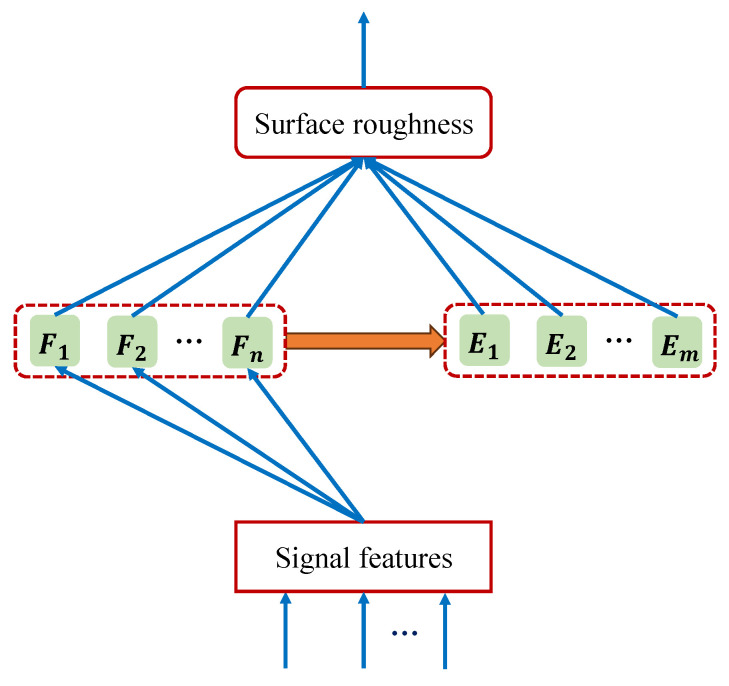
Basic architecture of a broad learning system.

A broad learning system (BLS) is an effective and efficient machine learning framework based on a random vector functional link neural network (RVFLNN) [23]. It is favored in industrial applications because of its depth-free network structure [24]. The basic architecture of a BLS is shown in Figure 1, which mainly consists of input layer, feature layer, enhancement layer, and output layer.

During the BLS training process, the original input of the input layer is first mapped to the feature layer through linear transformation. Then, the output of the feature layer is mapped to the enhancement layer through nonlinear transformation. Finally, the outputs of the feature layer and the enhancement layer are combined and connected to the final output layer. Notably, the connection weights between the input layer and the feature layer, as well as the connection weights between the feature layer and the enhancement layer, are randomly generated and remain unchanged throughout the learning process. However, the connection weights of the final output layer need to be calculated quickly using pseudo-inverse or ridge regression. Moreover, when the performance of a BLS does not meet the requirements, it can be extended through incremental learning, where only the connection weights of the newly added parts are recalculated without retraining the whole network. This eliminates the need for the tedious gradient descent method during training. Therefore, a BLS has good scalability and high training efficiency, making it more advantageous for small batches of training samples.

In this study, only the multiple-input single-output (MISO) case is considered. Assume that the sample dataset is

(1) $$\left\{\left(X,Y\right)|X\in R^{N\times D},Y\in R^{N\times 1}\right\}$$

where *N* is the number of all samples and *D* is the dimension of the feature. $X=\left(x_{1},x_{2},\cdots ,x_{N}\right)$ and $Y=\left(y_{1},y_{2},\cdots ,y_{N}\right)$ denote the input matrix of the sample and the corresponding output label vector, respectively. For convenience of expression, the feature layer and enhancement layer in the BLS architecture are regarded as hidden layers.

Assuming that the feature layer of a BLS contains *n* feature groups, and each feature group contains *k* feature nodes, then the output of the *i*th feature group is calculated as follows:

(2) $$F_{i}=\varphi_{i}\left(XW_{F_{i}}+\beta_{F_{i}}\right)\in R^{N\times k},i=1,2,\cdots ,n$$

where $\varphi_{i}$ is a linear transformation, $W_{F_{i}}$ and $\beta_{F_{i}}$ denote the randomly generated weight matrix and threshold term with appropriate dimensions, respectively.

Then, the outputs of all feature group nodes are combined as follows:

(3) $$F^{n}=\left[F_{1},F_{2},\cdots ,F_{n}\right]\in R^{N\times nk}$$

Next, the output $F^{n}$ of the feature layer is further mapped to the enhancement layer through nonlinear transformation for feature expansion. Assuming that the enhancement layer contains *m* enhancement groups and each enhancement group contains *l* enhancement nodes, the output of the *j*th enhancement group can be expressed as

(4) $$E_{j}=\psi_{j}\left(F^{n}W_{E_{j}}+\beta_{E_{j}}\right)\in R^{N\times l},j=1,2,\cdots ,m$$

where $\psi_{j}$ represents a nonlinear activation function, $W_{E_{j}}$ and $\beta_{E_{j}}$ denote the randomly initialized weight matrix and threshold term in the enhancement layer, respectively.

Similarly, the outputs of all enhancement group nodes in the enhancement layer can be combined as follows:

(5) $$E^{m}=\left[E_{1},E_{2},\cdots ,E_{m}\right]\in R^{N\times ml}$$

Finally, the outputs of the feature and enhancement layers are spliced, that is,

(6) $$H_{m}^{n}≜\left[F^{n}|E^{m}\right]\in R^{N\times L}$$

where L=nk+ml.

Therefore, the final output of a BLS can be expressed as

(7) $$\hat{Y}=H_{m}^{n}W_{m}^{n}$$

where $W_{m}^{n}$ denotes the connection weight matrix between the hidden layer and the final output layer, which can be quickly calculated using pseudo-inverse or ridge regression as follows:

(8) $$W_{m}^{n}={\left[\lambda I+{\left(H_{m}^{n}\right)}^{T}H_{m}^{n}\right]}^{-1}{\left(H_{m}^{n}\right)}^{T}Y$$

where λ is a nonnegative regularization parameter and *I* is the identity matrix with proper dimensions.

### 2.2. Virtual Sample Generation Scheme

In general, more comprehensive signal features are extracted from sensor monitoring signals to achieve online monitoring of surface roughness, which undoubtedly increases the feature dimension of the sample and affects the model prediction accuracy due to the existence of irrelevant, collinear, and redundant features. Therefore, it is first necessary to select or reduce the dimensionality of the extracted signal features, and then generate virtual samples with the help of a prediction model. In order to address the problem of small samples and high dimensionality in the ultra-precision cutting process, this paper designs a novel surface roughness virtual sample generation scheme, named PSOVSGBLS, to realize small sample enhancement of surface roughness.

The virtual sample generation process was regarded as a constrained optimization problem in [25], where PSO was combined with the extreme learning machine (ELM) prediction model to generate virtual samples. Since the output labels of virtual samples are determined by the predictions of the model constructed from a small number of original training samples, virtual samples are regarded as reasonable when the MAPE of the prediction results is less than 10% [26]. First, feature extraction and selection are performed on the process monitoring signals of the original training samples. Then, the original small samples are used to train the BLS model, and the optimal weight parameters are preserved. Finally, PSO is combined with the trained BLS to generate surface roughness virtual samples. The specific generation process is outlined below.

Assuming that the feature matrix of the original training sample is $X=\{x_{i}^{j}\in R|i=1,2,\cdots ,n,j=1,2,\cdots ,m\}$, where *n* is the number of training samples and *m* is the number of attributes or features in the training sample. The center of the *j*th attribute can be calculated as follows:

(9) $$x_{CL}^{j}=\frac{1}{n}\sum_{i=1}^{n}x_{i}^{j}$$

**Figure 2:**
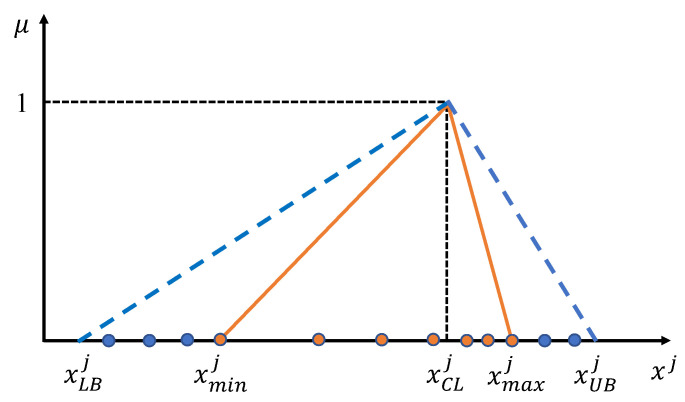
TMIE attribute domain expansion diagram.

Firstly, the information-expanded function based on triangular membership (TMIE) [25] is used to asymmetrically expand the attribute domain range of the selected features in the original training samples, as shown in Figure 2, where the abscissa indicates the observed values of the sample attributes, and the ordinate indicates the occurrence possibility of the observations.

As can be seen in Figure 2, the skewness of the triangle in the TMIE function is closely related to the number of samples on the left and right sides of each attribute center $x_{CL}^{j}$. This shows that the magnitude of the left and right skewnesses can be regarded as a measure of the asymmetry of the sample distribution. The left and right skewnesses of the *j*th attribute are calculated as follows:

(10) $$x_{SK_{L}}^{j}=\frac{x_{N_{L}}^{j}}{x_{N_{L}}^{j}+x_{N_{U}}^{j}+s_{p}}$$

(11) $$x_{SK_{U}}^{j}=\frac{x_{N_{U}}^{j}}{x_{N_{L}}^{j}+x_{N_{U}}^{j}+s_{p}}$$

where $x_{SK_{L}}^{j}$ and $x_{SK_{U}}^{j}$ are the number of samples less than and greater than the center in the *j*th attribute, respectively, and $s_{p}$ is used to fine-tune the skewness, which is set to 1 here. Thus, the upper and lower bounds of the expansion domain of the *j*th attribute can be defined as

(12) $$x_{LB}^{j}=x_{CL}^{j}-\frac{1}{x_{N_{U}}^{j}}\times \left(x_{CL}^{j}-x_{min}^{j}\right)$$

(13) $$x_{UB}^{j}=x_{CL}^{j}+\frac{1}{x_{N_{L}}^{j}}\times \left(x_{max}^{j}-x_{CL}^{j}\right)$$

where $x_{min}^{j}$ and $x_{max}^{j}$ denote the minimum and maximum values of the *j*th observed attribute, respectively.

Then, to generate more reasonable surface roughness virtual samples, PSO is used to optimize the minimum relative error between the predicted value of the BLS and the actual measured value to search for the optimal combination between the input features and output labels to serve as the surface roughness virtual samples. Mathematically, this process is described as follows:

(14) $$\left\{\begin{array}{c} \underset{y\in Y}{min}\left(100\times \left|\frac{y-\hat{y}}{y}\right|\right)\\ s.t.\left\{\begin{array}{c} x_{LB}^{j}\leq x^{j}\leq x_{UB}^{j}\\ \left|\frac{y-\hat{y}}{y}\right|\leq 0.1 \end{array}\right. \end{array}\right.$$

**Figure 3:**
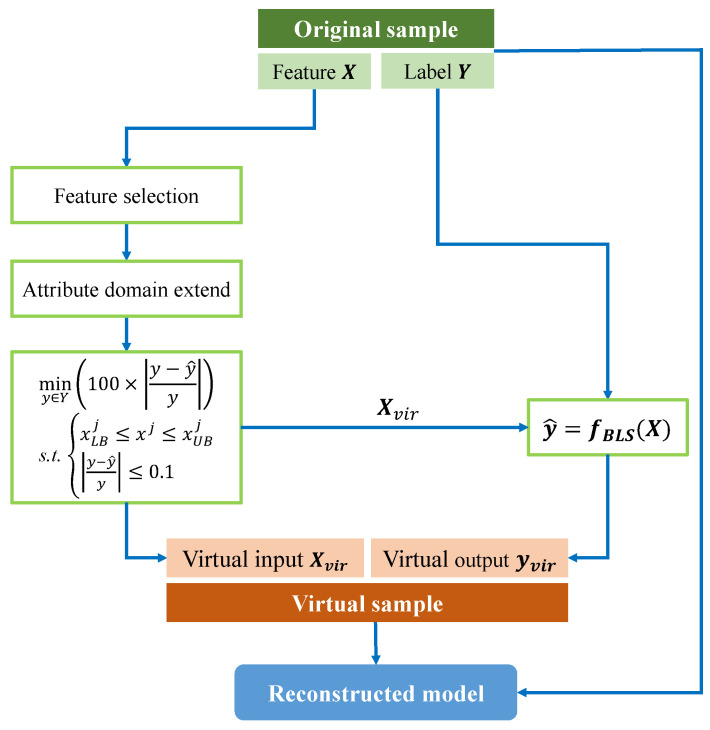
PSOVSGBLS architecture diagram.

Finally, the original training samples and the generated virtual samples are merged, and the BLS model is retrained to evaluate the reduction in prediction error before and after incorporating the virtual samples. The architecture of PSOVSGBLS is shown in Figure 3. In summary, the specific operation process of using sensor monitoring signals to generate surface roughness virtual samples can be briefly summarized as follows:

(1) Extract features from the monitoring signals during the cutting process and then select features. Use the original small samples to train the BLS model and save the optimal weight parameters.
(2) Extend the attribute domain of the selected optimal features using TMIE.
(3) Randomly generate the input features of the virtual sample within the upper and lower bounds of the sample attributes. Generate the output labels of the virtual samples using the trained BLS model from step (1). Set the number of virtual samples to be generated, and then use PSO to search for the best combination between input features and output labels. This combination results in a set of virtual samples considered reasonable representations of surface roughness.
(4) Merge the generated virtual samples with the original small samples. Then, retrain the BLS model and evaluate the performance of the prediction results.

## 3. Experimental Design

### 3.1. Design of Experiment

**Figure 4:**
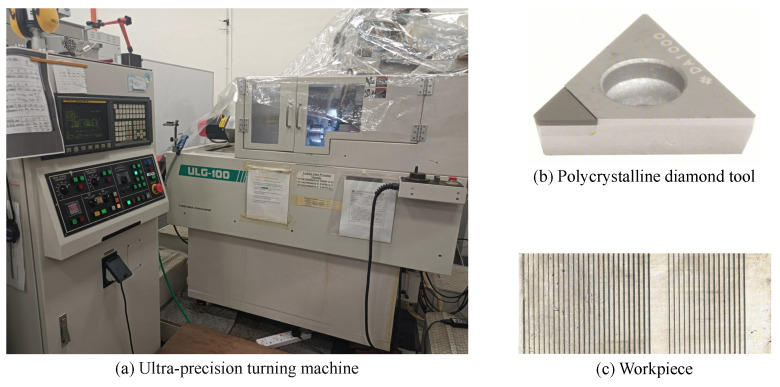
Experimental setup.

To verify the effectiveness of the proposed virtual sample generation scheme for surface roughness prediction in cutting processes, a groove-cutting experiment was carried out on a Toshiba ULG-100 ultra-precision turning machine. The workpiece material is aluminum alloy (Al 6061) with a dimension of 50 mm × 15 mm × 10 mm. The cutting tool is a polycrystalline diamond tool (NF-TPGW110308 DA1000) with a nose radius of 0.8 mm, as shown in Figure 4.

Before groove cutting, beeswax was first heated and melted on a custom-made tray, and the workpiece sample was placed in the beeswax and fixed on the tray after cooling. Then, the workpiece surface was trimmed to remove burrs and scratches to keep the surface smooth and flat. To this end, three repeated surface trimming cutting tests were carried out, during which the machine tool spindle speed *n* was 1200 rpm, the depth of cut $a_{p}$ was 3 μm, and the feed speed $v_{f}$ was 5 mm/min.

In this experiment, the tool was perpendicular to the workpiece surface and performed feed motion along the groove direction, akin to orthogonal cutting on the workpiece surface. A reasonable range of cutting parameters was established based on machine tool performance and machining experience. Two sets of full-factor cutting experiments were designed for model training and model testing, respectively, where each groove was cut under different combinations of cutting parameters. The cutting parameter combinations and measurement results used for model training and testing can be found in [16].

During the groove-cutting process, the spindle of the ultra-precision machine tool was stationary, while only the tool performed back-and-forth cutting along the cutting direction. The limit feed speed of the machine tool along the cutting direction was 450 mm/min. Therefore, the range of cutting depth $a_{p}$ was selected as [3 μm, 15 μm], and the range of feed speed $v_{f}$ was selected as [40 mm/min, 280 mm/min]. Air cooling was adopted throughout the cutting process. Since the hardness of the tool material significantly exceeded that of the workpiece material, the impact of tool wear on surface roughness was ignored, and instead, the impact of the cutting depth and feed speed on surface roughness was mainly considered.

### 3.2. Data Acquisition and Preprocessing

**Figure 5:**
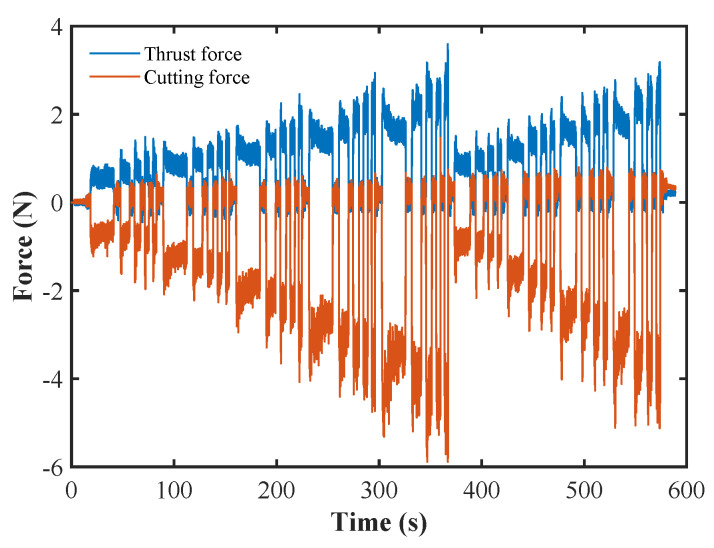
Original waveform diagram of the cutting force and thrust force.

To effectively monitor the dynamic changes of surface roughness, a three-component dynamometer (Kistler 9256C1) was installed on the tool holder of the ultra-precision machine tool. The cutting force $\left(F_{c}\right)$ and thrust force $\left(F_{t}\right)$ were collected using a 5051A-type charge amplifier with a sampling rate of 50 kHz. Figure 5 shows the original waveform diagrams of the cutting force and thrust force. During the acquisition process of the force signals, a center baseline drift occurred in the three-component dynamometer. In order to improve the data quality of the force signal, it was necessary to remove the drift and intercept the effective signal. Since each groove was processed under different combinations of cutting parameters and the required machining time varied, signals were manually intercepted at the cutting-in and cutting-out parts of each groove.

**Figure 6:**
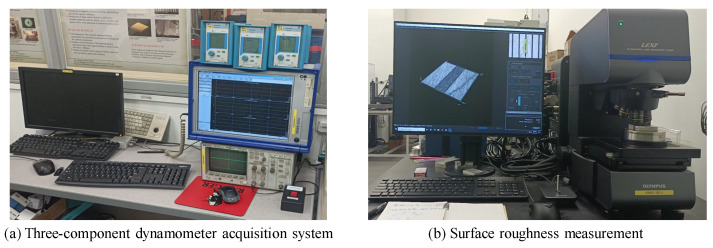
Force signal acquisition and surface roughness measurement.

After the cutting was completed, the workpiece was cleaned with alcohol, and the groove surface roughness was measured using a LEXT OLS5000 3D measuring laser microscope (Olympus, Japan). Sa was selected as the surface roughness evaluation index. To ensure the reliability of the surface roughness measurement results, measurements were taken at three different locations on each groove, and the average value was calculated as the final measurement result. During the surface roughness measurement process, the width of each groove was closely related to the cutting depth. In order to ensure consistent measurement conditions for each groove, a rectangular measurement area with a length of 1700 μm and a width of 125 μm was selected in the middle of each groove. The groove shapes were then removed, and finally, a Gaussian filter was applied for the measurement, with the S-filter set at 2 μm and the L-filter at 250 μm. The force signal acquisition and surface roughness measurements taken during the groove-cutting process are shown in Figure 6. Throughout the cutting process, there was no secondary clamping error between tool changes and workpiece handling.

Generally, time domain features or frequency domain features reflect the global characteristics of a signal but cannot capture the local characteristics of different frequency components of the signal. In the cutting process, the surface roughness monitoring signal usually exhibits non-stationary behavior. To address the limitations of the Fourier transform and reflect the time-domain and frequency-domain characteristics at the same time, this paper utilizes the wavelet packet transform (WPT) to extract the time-frequency domain features of the monitoring signal. First, the monitoring signal is divided into different frequency bands. Then, the changes in surface roughness are quantified by the percentage of energy in each frequency band relative to the total energy, serving as a feature.

## 4. Results and Discussion

In the groove-cutting experiment, there were a total of 41 valid sample data, of which 25 samples were used for model training and 16 samples were used for model testing. Each sample contained 32 time-frequency domain features. To ensure a fair comparison with the surface roughness sample generation results based on cutting parameters [16], 16 virtual samples of surface roughness were also generated using the force signal features. During the groove-cutting process, the thrust and cutting force signals of each sample were decomposed into four-layer wavelet packets using a db3 wavelet. Specifically, the thrust and cutting force signals were decomposed into 16 frequency bands, and the energy proportion of each frequency band was extracted as the feature vector.

Since different features have different dimensions and magnitudes, to eliminate the impact of the surface roughness virtual samples on the prediction results, it was necessary to normalize the original sample data of surface roughness during the generation process. The normalization process used in this article was as follows:

(15) $${\bar{x}}_{ij}=\frac{x_{ij}-min\left(x_{j}\right)}{max\left(x_{j}\right)-min\left(x_{j}\right)}$$

(16) $${\bar{y}}_{i}=\frac{y_{i}-min\left(y\right)}{max(y)-min(y)}$$

where $x_{ij}$ is the original data of the *j*th feature in the *i*th sample, $max(x_{j})$ and $min(x_{j})$ represent the maximum value and minimum value of the *j*th feature, respectively. Additionally, $y_{i}$ is the actual measurement value of the *i*th sample, max(y) and min(y) represent the maximum value and minimum value of the actual measurement results of all samples, respectively.

To evaluate the model prediction accuracy, the mean absolute percentage error (MAPE) was employed as the performance index, which is defined as follows:

(17) $$\mathrm{MAPE}=\frac{1}{N}\sum_{k=1}^{N}\left|\frac{y\left(k\right)-\hat{y}\left(k\right)}{y(k)}\right|$$

where *N* represents the number of samples, y(k) and $\hat{y}\left(k\right)$ denote the actual measurement result and the predicted result of the *k*th sample, respectively.

In addition, the error reduction rate ${\mathrm{ERR}}_{\mathrm{MAPE}}$ was used to represent the degree of reduction in the prediction error after incorporating the virtual samples, which is defined as follows:

(18) $${\mathrm{ERR}}_{\mathrm{MAPE}}=\frac{{\mathrm{MAPE}}_{\mathrm{before}}-{\mathrm{MAPE}}_{\mathrm{after}}}{{\mathrm{MAPE}}_{\mathrm{before}}}\times 100\%$$

where ${\mathrm{MAPE}}_{\mathrm{before}}$ and ${\mathrm{MAPE}}_{\mathrm{after}}$ represent the MAPE of the model prediction results before and after incorporating virtual samples, respectively. The larger the ${\mathrm{ERR}}_{\mathrm{MAPE}}$, the more pronounced the reduction in the model prediction error, indicating a more significant improvement in model prediction accuracy, and vice versa.

Signal features extracted from additional sensor monitoring signals often exhibit some irrelevance or redundancy. It is necessary to select important features to reduce model complexity and improve model prediction performance. Among them, the maximal information coefficient (MIC) can measure the degree of linear or nonlinear correlation between two variables and has the advantages of good universality and low complexity [27]. Therefore, in this work, MIC was used to select the important features of the sensor monitoring signals.

**Figure 7:**
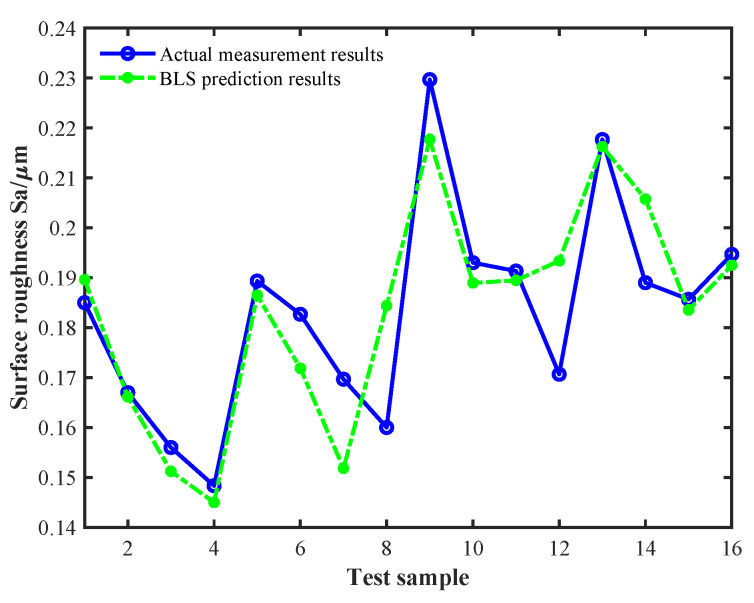
BLS prediction results for the test samples without the virtual samples.

**Figure 8:**
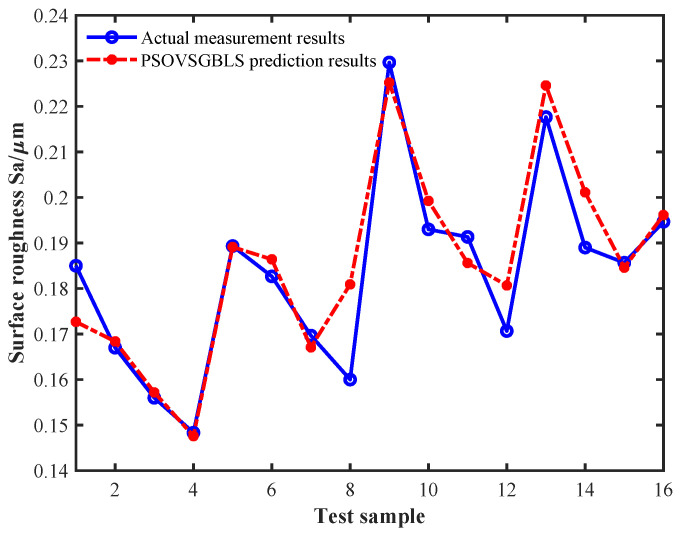
BLS prediction results for the test samples after incorporating the virtual samples.

First, the 32 features of the thrust and cutting force signals of the training sample were sorted from high to low based on the MIC and gradually accumulated as inputs to train the BLS model. The weighting parameters and optimal features were saved when the MAPE of the test samples reached the minimum, which was 4.68%. In this process, the selected optimal features were the energy proportions of the 5th, 6th, 7th, 11th, and 12th components of the wavelet packet corresponding to the thrust signal. The prediction results for the test samples are shown in Figure 7. Then, the selected five optimal features were used to extend the attribute domain using TMIE, and the PSO algorithm was used to search for the optimal combination between the virtual sample features and the output labels, where the output labels were predicted by the BLS model constructed from the original training samples. The initial parameter settings of the PSO algorithm during the virtual sample generation process are shown in Table 1. Finally, the 16 generated virtual samples were merged with the original 25 training samples, bringing the number of training samples to 41, while the test samples remained the same, and the BLS prediction model was retrained. Figure 8 shows the prediction results of the BLS model on the test sample after incorporating the virtual samples, and its MAPE became 3.15%. It can be seen that the prediction accuracy of the BLS model greatly improved after incorporating the generated virtual samples, and its error reduction rate reached 32.69%.

**Figure 9:**
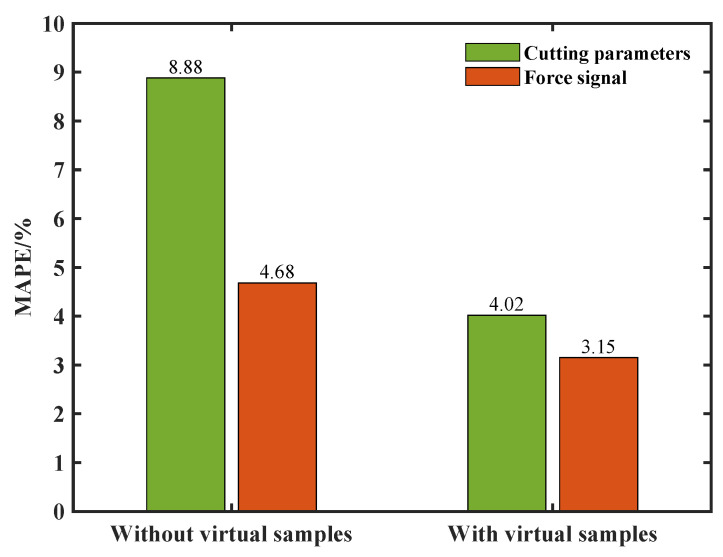
Comparison of prediction performance with and without virtual samples.

Moreover, compared with the surface roughness small sample enhancement results based on the cutting parameters proposed in [16], the use of force signal features demonstrated higher prediction accuracy with or without virtual samples, as shown in Figure 9.

In summary, the surface roughness virtual sample generation scheme proposed in this study has potential application prospects for addressing the small sample size problem in ultra-precision machining and can be extended to other machining fields. However, this work only selects the time-frequency domain features of the thrust and cutting forces in the groove-cutting process as model inputs and does not consider other signal features. Exploring how to fuse cutting parameters with signal features to construct a virtual sample generation model for generating reasonable virtual samples will be a promising research direction in ultra-precision smart machining processes. In addition, determining the appropriate number of virtual samples remains a challenging problem.

## 5. Conclusions

Aiming to address the small sample problem associated with surface roughness in ultra-precision machining, this work realizes small sample enhancement of surface roughness by generating effective virtual samples. In this article, a novel virtual sample generation scheme (PSOVSGBLS) for surface roughness is designed using force signals obtained during the machining process. Firstly, the wavelet packet transform is used to extract thrust and cutting force signal features from the original training samples, which are then sorted from high to low based on the MIC and gradually accumulated as inputs to train the BLS model for optimal feature selection. Secondly, the attribute domain of the selected optimal features is expanded using TMIE. Finally, PSO is combined with the trained BLS to generate surface roughness virtual samples. In addition, the effectiveness of the proposed scheme is verified through ultra-precision cutting experiments. The results demonstrate a significant improvement in the prediction accuracy of the BLS model after incorporating the virtual samples, with an error reduction rate of 32.69%. Furthermore, compared to cutting parameters, the force signals demonstrate higher prediction accuracy for surface roughness both before and after incorporating the virtual samples. In future work, virtual sample generation for surface roughness based on physics-constrained data is anticipated to be a promising area of exploration. 

## Figures and Tables

**Table 1:** Parameter settings for PSO.

Parameter	Particle Number	Maximum Iterations	$c_{1}$	$c_{2}$	$w_{\max}$	$w_{\min}$	Expected Fitness Value
**Value**	50	100	2	2	0.9	0.4	$10^{-8}$

## Data Availability

The data presented in this study are available on request from the corresponding author.
